# Virtual-Reality-Induced Visual Perturbations Impact Postural Control System Behavior

**DOI:** 10.3390/bs9110113

**Published:** 2019-11-12

**Authors:** Harish Chander, Sachini N. K. Kodithuwakku Arachchige, Christopher M. Hill, Alana J. Turner, Shuchisnigdha Deb, Alireza Shojaei, Christopher Hudson, Adam C. Knight, Daniel W. Carruth

**Affiliations:** 1 Neuromechanics Laboratory, Department of Kinesiology, Mississippi State University, Mississippi State, MS 39762, USA; snk128@msstate.edu (S.N.K.K.A.); ajt188@msstate.edu (A.J.T.); aknight@colled.msstate.edu (A.C.K.); 2 Department of Kinesiology and Physical Education, Northern Illinois University, DeKalb, IL 60115, USA; chill8@niu.edu; 3 Department of Industrial, Manufacturing, and Systems Engineering, University of Texas-Arlington, Arlington, TX 76010, USA; shuchisnigdha.deb@uta.edu; 4 Department of Building Construction Science, Mississippi State University, Mississippi State, MS 39762, USA; shojaei@caad.msstate.edu; 5 Center for Advanced Vehicular Systems (CAVS), Mississippi State University, Mississippi State, MS 39762, USA; chudson@cavs.msstate.edu (C.H.); dwc2@cavs.msstate.edu (D.W.C.)

**Keywords:** virtual reality, postural control, visual perturbations, postural stability behavior

## Abstract

Background: Virtual reality (VR) is becoming a widespread tool in rehabilitation, especially for postural stability. However, the impact of using VR in a “moving wall paradigm” (visual perturbation), specifically without and with anticipation of the perturbation, is unknown. Methods: Nineteen healthy subjects performed three trials of static balance testing on a force plate under three different conditions: baseline (no perturbation), unexpected VR perturbation, and expected VR perturbation. The statistical analysis consisted of a 1 × 3 repeated-measures ANOVA to test for differences in the center of pressure (COP) displacement, 95% ellipsoid area, and COP sway velocity. Results: The expected perturbation rendered significantly lower (*p* < 0.05) COP displacements and 95% ellipsoid area compared to the unexpected condition. A significantly higher (*p* < 0.05) sway velocity was also observed in the expected condition compared to the unexpected condition. Conclusions: Postural stability was lowered during unexpected visual perturbations compared to both during baseline and during expected visual perturbations, suggesting that conflicting visual feedback induced postural instability due to compensatory postural responses. However, during expected visual perturbations, significantly lowered postural sway displacement and area were achieved by increasing the sway velocity, suggesting the occurrence of postural behavior due to anticipatory postural responses. Finally, the study also concluded that VR could be used to induce different postural responses by providing visual perturbations to the postural control system, which can subsequently be used as an effective and low-cost tool for postural stability training and rehabilitation.

## 1. Introduction

Erect bilateral standing of the human body is characterized by the ability to maintain the center of mass (COM) of the body within the base of support (BOS) [1]. However, maintaining postural stability and equilibrium is challenging due to the arrangement of the COM in relation to the BOS, and it can be influenced by extrinsic or environmental factors as well as intrinsic or human factors. Thus, in order for humans to maintain balance, the perception, processing, and integration of sensory stimuli from the environment and periphery is required. Such sensory information is primarily derived from the visual, vestibular, and somatosensory receptors. These systems are hierarchically arranged with the most influential input provided by the visual system followed by the somatosensory and vestibular systems in a stable static standing position [1]. In instances where one sensory system is compromised or the sensory information is deemed inaccurate, a complex process of sensory organization and sensory reweighting maintains balance. Alterations to these sensory systems can affect the human body’s ability to maintain balance, especially when those changes are to the visual system. Multiple studies have demonstrated that manipulations to the visual environment alter postural control [2,3,4]. For example, visual cues displayed at a farther distance increased COP displacement, implying reduced balance and postural stability, than when visual cues were presented at a closer distance [5].

One popular visual manipulation is the “moving room” paradigm, which was first introduced back in 1974, in the context of motor development [6]. In this experiment, participants are asked to stand facing a wall that, unknowingly to the participant, moves toward them along the anterior–posterior axis. As a result, incongruent visual information is detected in relation to the body’s orientation; therefore, visual information is suppressed in favor of proprioceptive and vestibular information. Mechanically, this manipulation shifts the COM to the posterior edge of the BOS and destabilizes the upright standing posture. In order to maintain balance, a reflexive response is executed by the lower extremity muscles to compensate for the disruption. These compensatory postural responses (CPRs) are stimulated by sensory feedback resulting from the perturbation. Interestingly, prior knowledge of oncoming perturbations can influence the postural response that is executed. These proactive responses have been described in previous literature as anticipatory postural responses (APRs) [7,8,9]. Anticipating a postural disruption allows the central nervous system to adjust the overall bodily response to the perturbation through feedforward mechanisms to minimize the negative consequences that are associated with balance loss. Santos and team found that when participants anticipated postural perturbations, COP displacement in the anterior–posterior direction was smaller, implying greater balance and postural stability, when compared with unexpected perturbations, which demonstrates that prior knowledge changes reactionary postural responses. These findings suggest that APRs limit the need for large CPRs and highlight the importance of both APRs and CPRs in maintaining balance and postural stability under perturbed conditions [10,11].

Most of the previous literature examining reactionary and anticipatory postural control has utilized manipulations of the physical visual environment to disrupt balance. Recently, virtual reality (VR) has become a popular and cost-effective tool to provide balance disruptions. VR-based perturbations are typically provided by altering the virtual environment (VE). This is commonly done by unexpectedly rotating the visual field presented to the participant across the pitch, yaw, and roll principle axes [12,13,14]. Similar to the moving wall, this particular paradigm provides inaccurate visual sensory information and thus forces an increased reliance on the vestibular and somatosensory systems to maintain balance. Such sensory reorganization is the foundation for VR-based perturbation training and has demonstrated effectiveness in the rehabilitation of balance deficits in stroke and Parkinson’s disease [15,16,17,18].

Interestingly, within the current body literature, the roles of CPRs and APRs remain unclear when perturbations are provided in a VE. Given that VR has become a popular tool for balance and postural stability rehabilitation, it is imperative to understand how CPRs and APRs manifest when unexpected and expected visual perturbations to the postural control system are provided in a VE. Additionally, the usage of the moving wall paradigm has not been implemented in a VE; thus, the effects of VE postural disruptions remain undressed. It was hypothesized that the “moving wall” paradigm could be introduced in a VE and that expected or anticipated postural disruptions would result in better postural stability through feedforward adjustment.

## 2. Materials and Methods

### 2.1. Participants

Twenty healthy, recreationally active collegiate students were recruited and completed the study. The inclusion criteria comprised a physically active status based on the American College of Sports Medicine (ACSM) criteria for physical activity, which included a minimum of 3–4 days of aerobic exercise per week and 2 days of resistance training per week for at least the past 3 months [19]. The exclusion criteria comprised the presence of any recent visual, vestibular, neurological, or musculoskeletal disorders. Any individual having any physical difficulty with completing the study was also not included in the study. Additionally, all participants also completed a physical activity readiness questionnaire (PAR-Q) and a simulation sickness questionnaire (SSQ) (explained further under methods) in response to VE exposure, in which a score difference between before and after VE exposure was used to exclude the individual from the data analyses (explained further under results). Based on the initial recruitment inclusion and exclusion criteria, 20 participants were tested, and based on the SSQ criteria, one participant was excluded from the data analysis, giving a total sample size of 19 participants (age: 25 ± 5.6 years; height: 166.13 ± 11.1 cm; mass: 67.86 ± 12.4 kg). The study was approved by the University’s Institutional Review Board (IRB), and the data collection was done at the University’s Neuromechanics Laboratory.

### 2.2. Instrumentation

Participants’ static postural stability was measured with a force platform (AMTI AccuGait, Watertown, MA, USA). Required VEs were developed with Unity 3D and were delivered via an HTC Vive Pro (HTC America, Inc. Seattle, WA, USA) head-mounted display. A lobby environment and a closed-room environment were developed as the two VEs used in the study.

### 2.3. Study Design

The experimental procedures followed a repeated-measures design, in which each participant served as their own control and was tested at baseline (no visual perturbation) and following unexpected and expected visual perturbations. The same order of testing was used for all participants based on previous literature [20,21,22,23,24], which indicated the use of unexpected perturbations before expected perturbations, as exposure to the expected perturbations first might not allow true unexpected perturbations. However, the potential limitation of the preset testing order may have led to order effects or carryover effects; this is addressed in the limitations section of the manuscript.

### 2.4. Experimental Procedures

**Figure 1:**
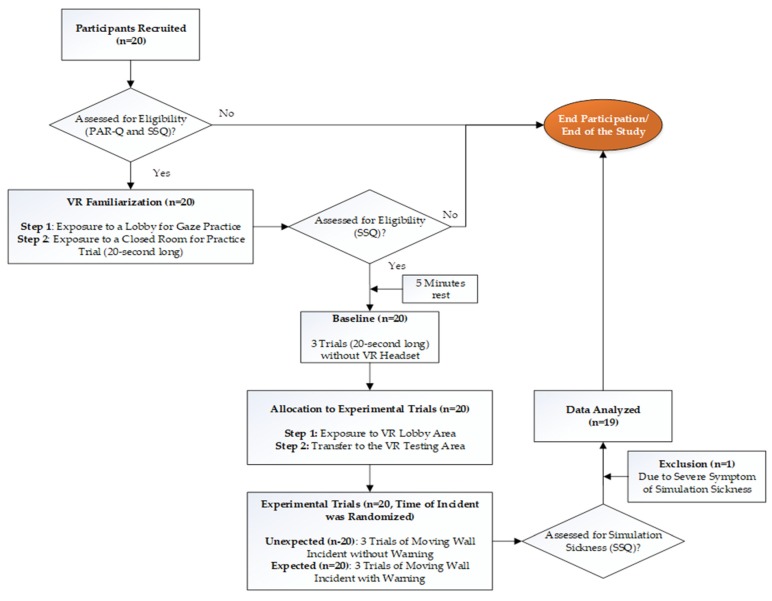
A flowchart of the experimental procedures followed for each participant.

**Figure 2:**
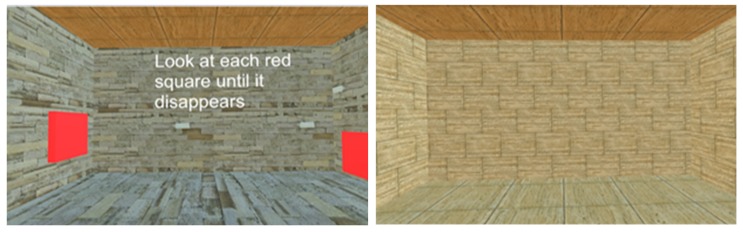
Virtual environments used in the study. Left: lobby environment, right: closed room environment.

The experimental procedures followed for each participant, from recruiting to completion of testing as well as data analysis, are explained in Figure 1. Upon arriving at the laboratory, the study protocol was explained to the participants, and written consent to participate in the study was obtained. To further assess eligibility for the study, a PAR-Q and an SSQ were given. Anyone with risk factors in the PAR-Q and/or an SSQ score > 5 was excluded from the study. Ahead of every data collection session, the force plates were calibrated in a static standing position for the postural stability tests, and the sampling rate of 100 Hz and duration of 20 s were set. Additionally, the force platform was zeroed using the “tare” function on the force platform NetForce software. The VR system was calibrated using the hand-held sensors to make sure the virtual environment was oriented in the correct direction and with the correct dimensions of the room and boundaries of the room. Following the collection of anthropological data, participants were familiarized with the examination protocol. Initial familiarization was done with the force plate; then, the participants were provided with a VR headset. During familiarization with the VE, participants were first exposed to the lobby area VE which was treated as a familiarization VE (Figure 2, left). This was done to familiarize them with VE as well as to practice their ability to use their gaze to complete given tasks. As the final phase of familiarization, they were exposed to the new closed room with the VE for 20 s, which was treated as the testing VE (Figure 2, right). During each VR task, corresponding instructions appeared on the screen (Figure 2, left), and additional verbal commands were given. Immediately upon completion of familiarization, the participants completed a second round of SSQ to assess their experience with VR exposure, and if the SSQ score > 5, the study was withheld.

**Figure 3:**
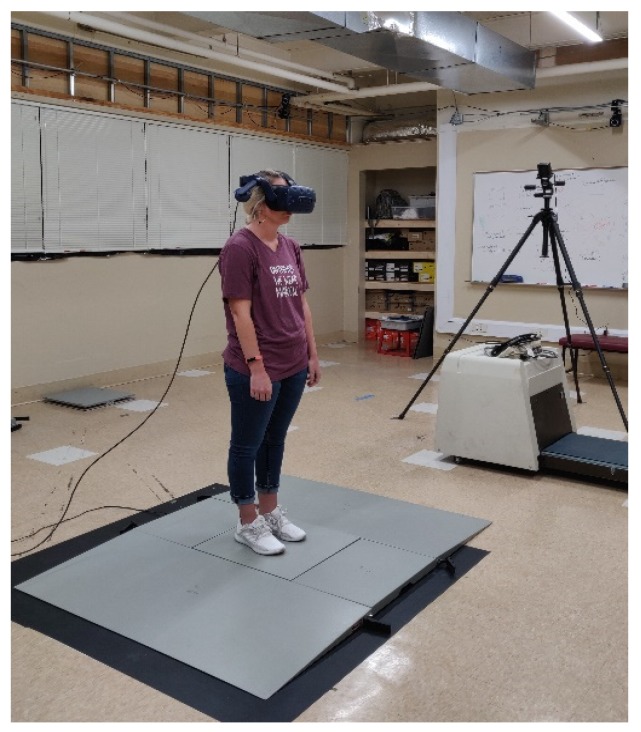
Participant being tested for postural stability wearing a virtual reality (VR) headset.

Following a five-minute rest after familiarization, three trials of baseline bilateral static postural stability were recorded in the eyes open condition with quiet standing, arms by side, eyes fixed at a specific point, and without the VR headset. Upon completion, participants were provided with the VR headset and were initially exposed to the lobby area VE (Figure 2, left), which was designed as a familiarization environment and as a transitionary environment before moving onto the testing VE. Moving to the testing VE was accomplished by participants using their gaze to make a box disappear (which was practiced during familiarization) to shift onto a new testing VE. Following disappearance of the box in lobby area, they were exposed to the new testing VE, which involved a closed room VE (Figure 2, right). Participants were advised to stand as erect as possible without moving and to look straight at the front wall of the room. Apart from these instructions, participants were not provided with any other information. An example of the participant set-up with the VR headset and force platform is shown in Figure 3. For all testing trials, the same investigator attempted to move the wall at three random times during the three trials within a 20 s time window to minimize the anticipation by participants. The initiation of the moving wall was based on a random time decided by the same investigator, and the result was that the front wall of the testing VE moved towards the participant without a prior warning. These three trials were considered as three unexpected wall movement trials. On completion of the unexpected trials, participants were exposed to three expected wall movement trials within the same 20 s time window, where the wall was moved following a warning and a countdown. The warning statement issued to all participants was that front wall would start to move on the “go” signal, which was provided as a three second countdown of three, two, one, and “go”. These trials were considered as expected wall movement trials. This marked the end of the study and participants were provided with a third round of SSQ and an experience survey. In an event of SSQ score > 5, the participants were monitored in the laboratory until they felt comfortable and were then dispersed.

### 2.5. Data Analyses

Kinetic data were analyzed with AMTI’s BioAnalysis software (Watertown, MA, USA). Based on COP excursions during the testing trials, medial–lateral displacement (average displacement along the X-axis), anterior–posterior displacement (average displacement along the Y-axis), 95% ellipsoid area, and average sway velocity were calculated and considered as the postural sway variables of interest. The raw data from the force platform were exported to BioAnalysis software from AMTI. The raw data were filtered using a preset fixed third analog filter and used for further data analysis using the balance analysis function to calculate the postural sway variables. Average COP sway displacements in the medial–lateral (M/L) and anterior–posterior (A/P) directions were calculated from the sum of absolute values for the initial M/L and A/P, subtracted from the average M/L and A/P COP values and divided by the number of data points from each postural stability testing trial (Equations (1) and (2)). The average COP sway velocity was calculated by dividing the total length of the COP path by the number of data points multiplied by the change in time over the duration of each postural stability testing trial (Equation (3)). The 95% ellipsoid area (cm^2^) represents an area of the ellipsoid based on the COP shifts in such a way that 95% of the data is within the ellipsoid and 5% is outside it [25], and subsequently, greater sway displacement, velocity, and area represent decreased balance and postural stability [23,24,26]. Descriptive statistics of participants’ responses to SSQ were calculated. Based on the results from the SSQ, one participant was excluded from the data analyses, as their SSQ after the VE exposure was greater than a score of five.

(1) $$\mathrm{Average}\;M/L\;\mathrm{sway}\;\mathrm{displacement}\;=\;\frac{\sum_{i=1}^{N}\left|x_{i}\;-\;AVG_{x}\right|}{N}$$

(2) $$\mathrm{Average}\;A/P\;\mathrm{sway}\;\mathrm{displacement}\;=\;\frac{\sum_{i=1}^{N}\left|y_{i}\;-\;AVG_{y}\right|}{N}$$

(3) $$\mathrm{Average}\;\mathrm{sway}\;\mathrm{velocity}=\;\frac{L}{N*\;∆t}$$

where $\left(L\right)=\;\sum_{i=2}^{n}\sqrt{{\left(x_{i}\;-\;x_{i-1}\right)}^{2}+{\left(y_{i}\;-\;y_{i-1}\right)}^{2}}$

### 2.6. Statistical Analyses

Postural sway variables of interest were analyzed with a one-way repeated measures analysis of variance [ANOVA] between the baseline, unexpected wall movement, and expected wall movement conditions. Any significant main effect was further analyzed with post-hoc pairwise comparisons using the Bonferroni correction factor. Statistical analyses were done with SPSS software v.25 (IBM SPSS, Armonk, NY, USA) with an a priori alpha level of 0.05.

## 3. Results

**Figure 4:**
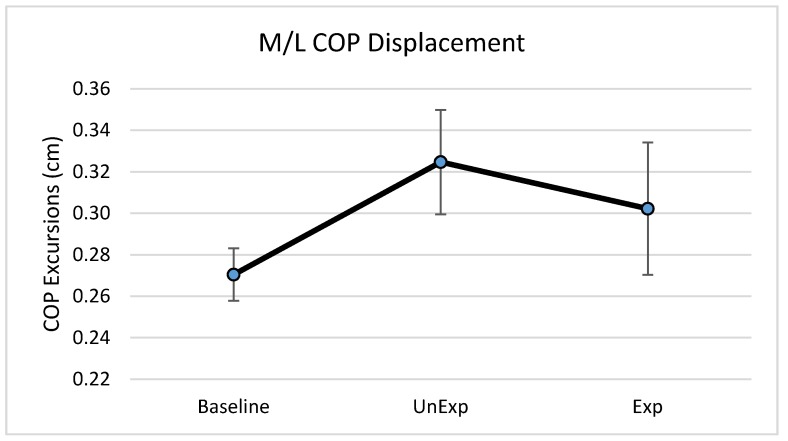
Center of pressure (COP) medial–lateral excursions (cm) during baseline, unexpected (UnExp) moving wall, and expected (Exp) moving wall conditions. Bars represent standard errors.

**Figure 5:**
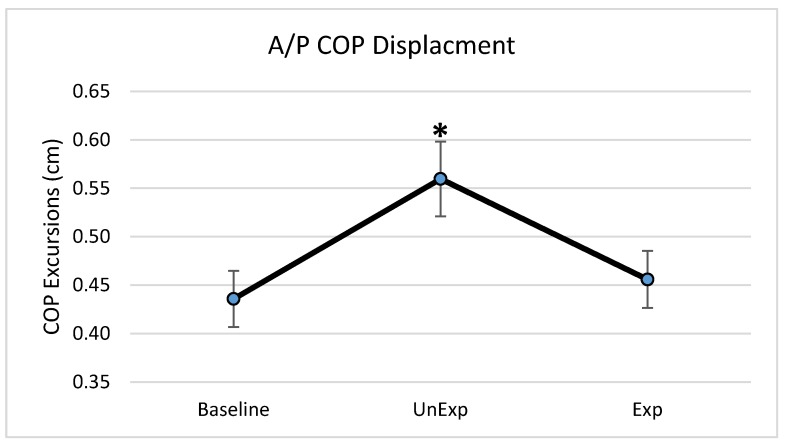
Center of pressure anterior–posterior excursions (cm) during baseline, unexpected (UnExp) moving wall, and expected (Exp) moving wall conditions. Bars represent standard errors. * Represents a significant difference at *p* < 0.05 compared to the baseline condition.

**Figure 6:**
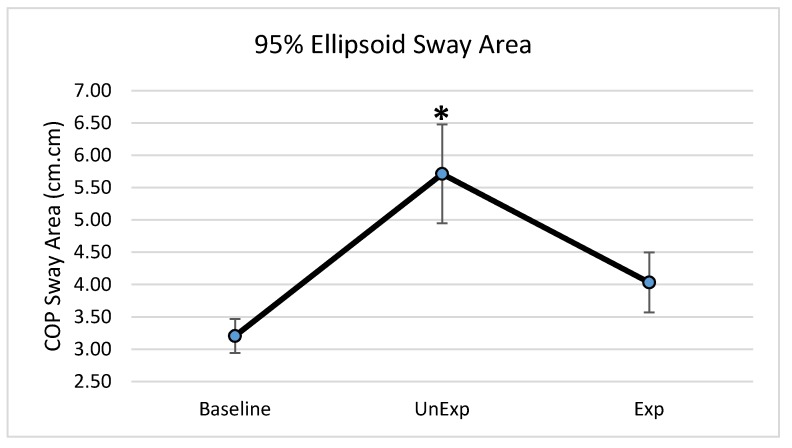
Center of pressure of the 95% ellipsoid sway area (cm^2^) during baseline, unexpected (UnExp) moving wall, and expected (Exp) moving wall conditions. Bars represent standard errors. * Represents a significant difference at *p* < 0.05 compared to the baseline condition.

**Figure 7:**
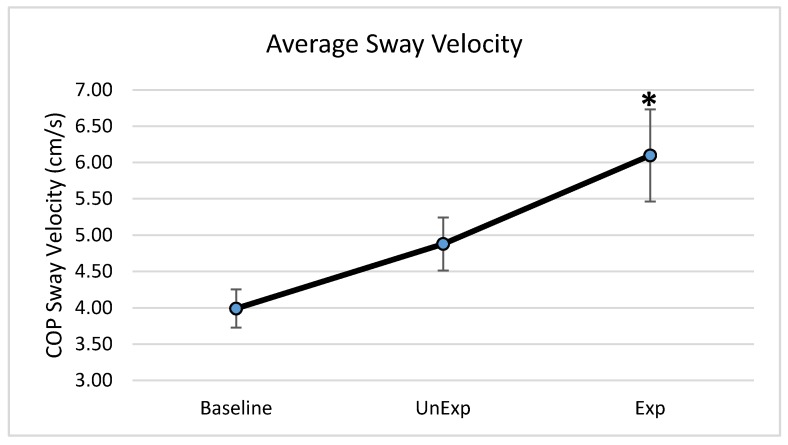
Center of pressure average sway velocity (cm/s) during baseline, unexpected (UnExp) moving wall, and expected (Exp) moving wall conditions. Bars represent standard errors. * Represents a significant difference at *p* < 0.05 compared to the baseline condition.

Based on the results from the SSQ, one participant was excluded from the analyses in response to VE exposure. Hence, 19 participants, which included nine males and ten females, were used for the analyses (age: 25 ± 5.6 years; height: 166.13 ± 11.1 cm; mass: 67.86 ± 12.4 kg). The main effects of the repeated-measures ANOVA were analyzed, and the pairwise post-hoc analyses with a Bonferroni correction were performed if a significant main effect was found. The repeated-measures ANOVA revealed a significant main effect between the testing environments for the postural sway variables of anterior–posterior displacement (F (2,36) = 6.903, *p* = 0.003, partial eta squared = 0.277); 95% ellipsoid sway area (F (2,36) = 8.627, *p* = 0.001, partial eta squared = 0.324), and average sway velocity (F (2,36) = 9.777, *p* < 0.0001, partial eta squared = 0.352), but not medial–lateral displacement (Figure 4). Pairwise comparisons of significant main effects revealed that for both anterior–posterior displacement (Figure 5) and the 95% ellipsoid sway area (Figure 6), the unexpected moving wall induced a significantly greater postural sway compared with baseline, with no differences between baseline and expected moving wall conditions. For the average sway velocity, pairwise comparisons revealed that the expected moving wall induced significantly greater postural sway compared with baseline, but not compared with the unexpected moving wall (Figure 7).

## 4. Discussion

The purpose of this study was to determine if visual perturbations that impact postural stability, could be introduced through VE and to determine the impact of VR-generated unexpected and expected visual perturbations on various postural stability measures. This study found differences in postural stability parameters between baseline and unexpected conditions as well as between baseline and expected visual perturbation conditions, suggesting differences in postural control strategies when a postural disruption is unanticipated vs. anticipated. More specifically, postural stability quantified by anterior–posterior COP displacement and the 95% ellipsoid sway area was significantly higher in the unexpected perturbation condition compared with the baseline, suggesting decreased postural stability when visual perturbations were unanticipated. During the expected perturbation condition, postural sway displacement and sway area were not significantly different from the baseline no-perturbation conditions, suggesting that postural stability was better and similar to no perturbation conditions when the visual perturbations were anticipated. However, during the expected perturbations, even though the postural sway displacement and area were significantly lower than following the unexpected perturbation and similar to baseline, individuals exhibited a significantly higher sway velocity compared to in unexpected and baseline conditions, suggesting a rapid postural behavior response to significantly minimize postural sway displacement and the postural sway area. Evidence of both CPRs and APRs during unexpected and expected visual perturbations was identified during this “virtual moving wall” experiment. Results from the study also suggested that VE using VR can be used as an effective method to induce visual perturbations.

The role of visual feedback for postural control and stability has been well established, in that the presence of visual feedback promotes postural stability and the absence of visual feedback is detrimental to postural stability, especially if there are associated vestibular and somatosensory feedback abnormalities [1,3,6,13]. Hence, with no visual perturbations, the baseline data provided the postural behavior when all three sensory system were providing appropriate sensory feedback for postural stability and equilibrium maintenance. Subsequently, postural stability was compromised with significantly higher postural sway when visual feedback was altered, especially with unexpected visual perturbations, which required CPRs to correct perturbations to postural stability. Previous studies have also suggested that anticipation changes the pre-perturbation activity of lower extremity muscles and moves the body’s center of mass in the direction of the oncoming perturbation [10]. This study found when perturbations were anticipated, COP displacement was lower than when it was unexpected and was similar to the baseline no-perturbation condition. The significantly lowered COP displacement during the expected visual perturbations suggests that postural behavior with APRs occurs to minimize excursions of the COM and COP trajectories [27]. Prior to the oncoming perturbation, the APRs move the COM in the direction of perturbation, which arranges the lower extremity body segments in a mechanically advantageous position to maintain an upright posture. Therefore, at perturbation onset, the displacement of the COM is smaller, resulting in lower COP displacements and better body balance. These pre-perturbation corrections are the result of the central nervous system preparing for the direction and magnitude of the disruption, which renders a more efficient response. In the case of this study, the participants were made aware that the VE would be disrupted; thus, adequate feed-forward corrections were implemented to counteract the perturbation, resulting in lower displacement of the COP in the anterior–posterior direction, which is also the direction of the moving virtual wall. Without the prior knowledge of an oncoming perturbation, the ability to maintain upright stance is more challenging; thus, the use of any CPR is based on sensory information derived from the postural disruption and cannot be as accurate as an APR.

In contrast to the findings of anterior–posterior displacement, no differences were found in medial–lateral displacement between the expectation conditions. The perturbations in the current study were provided along the anterior–posterior axis (wall moving directly at the participant), suggesting that the participants shifted their COM in the anterior direction with the corrective response. With the perturbation exclusively occurring on the anterior–posterior axis, medial–lateral stability remained intact during the postural disruption, resulting in no significant differences in displacement between baseline, unexpected, or expected visual perturbation conditions.

This study also found that average sway velocity was significantly higher in the expected condition compared with the unexpected condition. Typically, increased postural sway velocity is associated with poor balance and postural instability [28,29]. Pairing these results with the significantly smaller postural sway displacements in the expected condition compared with the unexpected condition suggests that the observed results could be due to a postural control behavioral response associated with APRs that elicits a faster sway velocity but not a larger sway displacement. The findings of the current study are in contrast with those of Santos and team, who demonstrated lower postural sway velocities when the perturbation was expected. However, the findings of the current study may suggest increased co-contraction of the distal muscles surrounding the ankle joint. Warnica and colleagues [30] found that as the co-contraction index of the ankle musculature increased, COP velocity increased in parallel, suggesting that increased rigidity around the ankle created higher sway velocities in response to balance perturbations [30]. Co-contraction is also seen as an attempt to maintain postural stability, increase balance control, and improve ankle stability in healthy adults [31], especially during the anticipation of perturbation when the postural control system is challenged [32]. Thus, in the current study, when the virtual perturbations were expected, the ankle musculature may have increased in rigidity by co-contracting distal ankle muscles, thus leading to increased COP velocities. The observed increased postural sway during the expected condition suggests that increased co-contraction of the ankle musculature occurred to minimize postural sway displacements and improve postural stability. However, this assertion needs to be confirmed by future studies utilizing electromyography.

The 95% ellipsoid sway area is a common method for quantifying the oscillatory activity during upright standing and is considered to be representative of postural steadiness, variability, and the energetic cost of postural control [33,34,35]. In the unexpected condition, the 95% ellipsoid sway area was significantly increased compared to the baseline and expected conditions, suggesting an increase in postural instability and an inefficient postural response. In the unexpected condition, CPRs are primarily used to maintained balance and are executed within a short time window based on limited sensory information. This short latency for sensory detection during such unanticipated visual perturbations renders a CPRs that is energy inefficient and highly variable, leading to a significantly higher 95% ellipsoid sway area during the unexpected visual perturbation condition. Conversely, APRs are tailored towards efficiency by accounting for the direction and magnitude prior to the perturbation [9,36] and create an energy-saving response, with low variability, as is evident from the significantly lower and similar to baseline condition values for the 95% ellipsoid sway area during the expected visual perturbation condition.

There are several limitations to the study that need to be considered, especially during comparison of the observed results to other similar research or with real-world applications. The potential limitation to the preset testing order of baseline, unexpected, and expected could have influenced the results due to order effects or carryover effects of testing, and this needs to be taken into account when interpreting results. However, the unexpected perturbations were provided in randomized intervals and the exposure of expected perturbations initially might not provide a true unexpected perturbation. The observed results of postural stability behavior were due to a VE in a closed room environment resembling the physical environment from the Lee and Aronson (1974) [6] experiment, and application of the current results with other VEs should be done with caution. Additionally, the observed results were due to a standardized preset wall movement velocity, while faster or slower velocities can induce different postural stability behavior results. Other limitations included the testing of only healthy adults in comparison to using elderly or clinical populations with neurological or musculoskeletal problems. As aging and diseased states impact postural responses and stability, the study sample was limited to healthy adults. Additional analysis of the postural control system using 3D motion capture to quantify COM and lower extremity kinematics and an electromyography system to quantify the co-contraction index of ankle muscles could improve the understanding of the postural control system’s behavior. Finally, the impact of VR use and exposure can be different in different individuals and different populations. While all participants were familiarized with the VE with VR, the current study involved an acute exposure to VE, and future studies should also attempt to identify the effects of chronic exposure to VE, especially with conflicting visual perturbations to the postural control system. The current study also used the SSQ before and after VR exposure to identify any abnormal consequences due to VR exposure to minimize variability. However, the usage of VR in different populations, especially among aging populations, and especially with visual perturbations, should be undertaken with extreme caution to avoid any fall risk. Finally, the use of treadmill-induced somatosensory system perturbations has already proven to be an efficient training method for postural stability [37], where the use of VR to provide visual perturbations over a set training period could act as the basis for an efficient, yet low cost, rehabilitation tool for individuals with postural stability deficits.

## 5. Conclusions

In conclusion, this study demonstrated decreased postural stability following unexpected visual perturbations, which was evident through significantly increased postural sway displacement and sway area, compared with the expected visual perturbation condition. However, this was achieved with increased postural sway velocity, suggesting that postural control responses were modified under the expected visual perturbation condition, similar to that seen with other paradigms. Additionally, the study also confirmed that postural perturbations could be induced through visual systems in a VE using VR. These findings build on the current body of literature, demonstrating that APRs are more efficient and lead to better postural control outcomes compared with using only CPRs and can be incorporated into balance rehabilitation programs.
